# Individual quality of life in spousal ALS patient-caregiver dyads

**DOI:** 10.1186/s12955-020-01551-5

**Published:** 2020-11-23

**Authors:** Miriam Galvin, Tommy Gavin, Iain Mays, Mark Heverin, Orla Hardiman

**Affiliations:** 1 Academic Unit of Neurology, Trinity College Dublin, Dublin, Ireland; 2 National ALS/MND Centre, Beaumont Hospital, Dublin, Ireland

## Abstract

**Background:**

Quality of life is a basic goal of health and social care. The majority of people with Amyotrophic Lateral Sclerosis (ALS) are cared for at home by family caregivers. It is important to recognize the factors that contribute to quality of life for individuals to better understand the lived experiences in a condition for which there is currently no curative treatment.

**Aim:**

To explore individual quality of life of people with ALS and their informal caregivers over time.

**Methods:**

Over three semi-structured home interviews, 28 patient-caregiver dyads provided information on a range of demographic and clinical features, psychological distress, caregiver burden, and individual quality of life. Quality of life data were analysed using quantitative and qualitative methods with integration at the analysis and interpretation phases.

**Results:**

Individual Quality of Life was high for patients and caregivers across the interviews series, and higher among patients than their care partners at each time point. Family, hobbies and social activities were the main self-defined contributors to quality of life. The importance of health declined relative to other areas over time. Friends and finances became less important for patients, but were assigned greater importance by caregivers across the illness trajectory. Psychological distress was higher among caregivers. Caregiver burden consistently increased.

**Conclusion:**

The findings from this study point to the importance of exploring and monitoring quality of life at an individual level. Self-defined contributory factors are relevant to the individual within his/her context. As an integrated outcome measure individual quality of life should be assessed and monitored as part of routine clinical care during the clinical encounter. This can facilitate conversations between health care providers, patients and families, and inform interventions and contribute to decision support mechanisms. The ascertainment of self-defined life quality, especially in progressive neurodegenerative conditions, mean health care professionals are in a better position to provide person-centred care.

## Key implications

If Quality of life is conceptualised as being unique to individuals, it cannot be adequately assessed using standardised measures only.

Contributors to life’s quality are re-evaluated and reconfigured as patients and their care partners adapt to changing functional, material and emotional contexts.

Palliative and supportive care services should aim to support the factors identified to help maintain life quality as long as is practicable.

## Introduction

Amyotrophic Lateral Sclerosis (ALS) also known as Motor neurone disease (MND) is a progressive and terminal neurodegenerative illness. Primarily involving the motor system, it is a multisystem disease impacting physical and verbal functioning, with up to 40% of patients presenting with cognitive and behavioural impairment. There is currently no cure, limited treatment, and death usually occurs within 3 years from symptom onset [1]. A palliative approach, focused on symptom management and preservation of quality of life, is recommended from the time of diagnosis [2]. People with ALS/MND are primarily looked after in their own home by informal caregivers, usually family or friends. Informal caregivers are an important component in the ALS care provision system, enabling patients to remain at home rather than going to a care facility [3]. Caregivers continuously adapt to symptom management and changes in lifestyle and simultaneously deal with the worry, fear and the emotional impact of watching the deterioration of the patient [4]. Best practice guidelines in ALS care emphasize the inclusion of family/significant others in patients’ care [5] and the incorporation families closely into the decision-making process. We believe that it is important to assess the factors which people feel are important in their lives and monitor these, over time as part of clinical care. The ascertainment of self-defined contributions to life quality mean health care professionals will be in a better position to provide person-centred care.

### Quality of Life

Quality of Life (QoL) is regarded as a basic goal of health and social care. QoL has different philosophical, political and health-related designations [6, 7]. It is determined by health-related and non-health related factors. As a multidimensional phenomenon, it requires an integrated approach to its conceptualization, which in turn determines the choice of assessment approaches. The use of both individual assessments and standardized measures capture complementary facets that are considered important in life [8].

Studies have shown that patient quality of life is maintained as ALS/MND progresses [9], with evidence of psychological adaptation to deteriorating function [10, 11]. Important life factors move from those dependent on physical function to those that are not e.g. social, spiritual, and existential [12, 13]. Patients with ALS often have high QOL [14], continuing throughout the disease due to shifting expectations and to reprioritization of factors contributing to QOL. Quality of life of caregivers is found to worsen as the disease progresses and care demands increase [3]. The elements of QoL change over time and in response to changing circumstances [15]. Accommodation to illness might explain changing values and the conceptualisation of QoL [16]. What people perceive as of value in their lives may change, affecting self-evaluation of quality of life and the importance of its component domains [14, 17].

Assessment of Individual Quality of Life (IQoL) is based on what someone considers to be personally important to them [18]. Individualised measures are designed to increase respondents’ discretion in selecting the areas of life (domains) that are most important and/or determining the relative importance of these domains [19]. As such the contributing factors are not the same for everyone and are individually assessed. The expression of non-predefined domains differs from the more usual predetermined biomedical model of QoL assessment, the latter may evaluate aspects of QoL that have little relevance to their individual concerns [20].

This analysis uses an individual level approach to explore quality of life and the self-identified life domains contributing to it, in Irish ALS patient-caregiver dyads using the Schedule for the Evaluation of Individual Quality of Life (SEIQoL-DW) [18]. SEIQol-DW is acceptable for use in ALS in terms of its practical feasibility, internal validity and consistency reliability in this patient group [21]. Moons et al. (2004) suggest that SEIQol-DW is not a measure of QoL but found to be a valid and reliable instrument to explore determinants for patients’ quality of life [22].

The aim of this study is to explore the factors that determine individual quality of life over time for people with ALS and their informal caregivers The domains that are perceived to be important are anchored in the person’s individual experience and have been shown previously to change over time. This work is novel in that it both explores these domains as the disease progresses, and explores their temporal patterning as reported by patients and their caregivers.

Care of people with ALS largely takes place in the community, and family members are key figures in informal caregiving. Studies show a high concordance between the well-being of the patient and that of the caregiver, indicating that a reduced well-being of the caregiver can negatively impact the wellbeing of the patient [23, 24]. Thus, a focus on quality of life, and the areas of life that contribute to the wellbeing of both patient and caregiver [25] is essential to ensure best practice.

Health care professionals should recognize that the needs and goals of patients and caregivers differ [26]. We believe that it is important to assess the factors which people feel are important in their lives and monitor these, over time as part of clinical care. The ascertainment of self-defined contributors to life quality, especially in progressive neurodegenerative conditions, means health care professionals will be in a better position to provide person-centred care.

This is the first longitudinal, integrated analysis of individual quality of life for people with ALS and their care partners.

## Material and methods

### Research settings and participants

As part of ongoing longitudinal research, patients and their associated primary informal caregivers attending the National ALS Centre in Dublin, were consecutively invited to participate. The National ALS Centre in Beaumont Hospital treats approximately 80% of all ALS/MND patients in Ireland.

Over the course of 18 months during 2013–2015, patients and their caregivers were approached by members of the research team (IM, MG), and given information about the study. A caregiver in this study is defined as someone who provides informal (unpaid) care and has been identified by the patient to the treating clinical team, as his/her main informal caregiver. Written informed consent was obtained from all participants. Patients’ clinical details were available through the National ALS Register, for which they had consented to inclusion of their codified clinical and demographic data.

Inclusion criteria: diagnosis with ALS, attending at National ALS Centre, an identified main informal (unpaid) caregiver, aged 18 years and over.

Exclusion criteria: not diagnosed with ALS, caregiver from public or private agencies, patient with no informal caregiver, under 18 years of age, no provision of informed consent.

Speech impairment or difficulty with writing were not exclusion criteria in this study. For those patients with bulbar symptoms, the participant was offered assistance with the tasks, to indicate his/her preference by pointing to cue cards and given the opportunity to respond in writing or by eye-gaze technology if writing was affected.

There was no sample size determined for this study. We were not attempting to generalise findings or make inferences about a population. We aimed to assess self-defined individual quality of life for patients and their care partners at three interviews over the course of 18 months. Of those recruited to this study 56 people completed SEIQoL-DW over that time period. Ethical approval for this study was received from Beaumont Hospital Ethics (Medical Research) Committee (REC REF 12/84) and the Research Ethics Committee, Trinity College Dublin.

### Measures and data collection

Data were collected during semi-structured home interviews by researchers attached to a multidisciplinary clinic (IM, MG). The interviews were conducted with the patient and caregiver separately, at baseline and on two further occasions at 4–6 month intervals from 2013 to 2015.

Patients were categorised according to the MiToS functional staging system (0–5) [27], higher scores indicating disease severity, and a proxy of long-term outcome [28]. Cognitive and behavioural impairment was assessed using Edinburgh Cognitive and Behavioural ALS Screen (ECAS) [29] and the Beaumont Behavioural Inventory (BBI) [30] respectively. Caregivers were asked to indicate the number of hours care they provided on average per week.

Psychological distress and quality of life were assessed for patients and caregivers, with burden an additional assessment for caregivers.

#### Psychological distress

The Hospital Anxiety and Depression Scale (HADS) [31] is designed to assess anxiety and depression. HADS-T is the sum of two subscales for anxiety and depression and is an estimate of general psychological distress. A HADS –T cut off score of 12 indicates probable psychological distress [32].

#### Quality of life

Administered as a semi-structured interview the Schedule for the Evaluation of Individual Quality of Life (SEIQoL-DW) [18] respondents nominate what they consider to be the five most important areas of their life (cue/domain), their current level of satisfaction and then the relative importance of each of them. The SEIQoL-DW scores are person-specific [10], an overall SEIQoL index QoL score (SIS) is a summary score, generated by multiplying each cue’s weight by its corresponding satisfaction level, and summing the products across the 5 life areas [18]. The SEIQOL Index score ranges from 0 (worst possible QoL) to 100 (best possible QoL). Data collected on the five areas of life nominated, their relative importance to each other and the SIS scores are used in this analysis.

The SEIQoL-DW is of value in identifying factors which contribute to the well-being of an individual with ALS, however SEIQoL index scores may not reflect aggregate QoL of groups of patients with ALS [33]. Further detail about the administration of SEIQoL-DW is in Appendix A.

#### Caregiver burden

The Zarit Burden Interview [34] assesses caregiver self-reported burden and the impact of caregiving on their lives. The higher the total score (0–88), the higher the level of perceived burden. A ZBI cut-off score of 24 and above, indicates high burden [35].

HADS, ZARIT and SEIQOL-DW have all been used to assess psychological distress, caregiver burden and quality of life respectively in previous ALS research [4, 11, 33, 36, 37, 38],

## Analysis

Descriptive statistics describe the demographic and clinical characteristics of the participants, with bivariate and multivariate analyses (i.e. t-tests, ANOVA, and non-parametric equivalents) as relevant. During the interview, SEIQoL-DW responses were manually recorded by researchers.

Within a realist framework in an analysis of content, two coders (MG, TG) independently coded the respondent-identified life domains into categories based on their descriptions [39]. A coding frame was developed, differences were consensually reconciled. An audit trail was created noting coding decisions over the course of the study. The codes and categories were simultaneously quantitized [40] for integrated analysis.

The software used during this mixed methods analysis were; Microsoft Excel, IBM SPSS and QSR NVivo.

## Results

The SEIQoL-DW was administered over the course of 12–18 months, and participant attrition occurred at different timepoints. Reasons for non-completion included research fatigue, illness burden and progression. The number of dyads completing the assessment at each interview are shown in Table 1.

**Table 1:** Completion of SeiQoL-DW at interviews

		***Number of Dyads***
Interview 1	Dyads T1	77
Interview 2	Dyads T1 & T2	43
Interview 3	Dyads T1, T2 & T3	29^a^

Note: ^a^ One Dyad was removed from analysis due to poor data quality

77 dyads completed one SEIQoL assessment, 43 completed two and 28 dyad dyads completed three SEIQoL-DW assessments.

Patient and caregiver characteristics are summarised and presented in Tables 2, 3 and 4.

**Table 2:** DYAD characteristics

	***Patients (n = 28)***		***Caregivers (n = 28)***	
	n	%	n	%
***Sex***				
Male	19	67.9	9	32.1
Female	9	32.1	19	67.9
***Age (years) at first interview***				
Mean	61.8		60.6	
Standard deviation	8.8		8.4	
***Relationship to the patient***				
Spouse/partner	28		28	
***Live with patient***				
Yes	–		28	100
***Site of onset***				
Bulbar	7	25.0	–	
Spinal	20	71.4	–	
Respiratory	1	3.6	–	
***Time from Diagnosis to first Interview (months)***				
Mean	23.2		–	
Standard deviation	28.8		–	
Median	17.5		–	
Range	(1.5–136.2)		–

**Table 3:** Patient characteristics

	Patient					
	Interview 1		Interview 2		Interview 3	
***MITOS stage***	**n**	**%**	**n**	**%**	**n**	**%**
0	21	75.0	18	64.3	13	46.4
1	6	21.4	8	28.6	11	39.3
2	–	–	2	7.1	2	7.1
3	–	–	–	–	–	–
Not available	1	3.6	–	–	2	7.1
***Cognition (ECAS)***						
Normal	21	75.0	20	71.4	18	64.3
Non-normal	6	21.4	7	25.0	9	32.1
Not Available	1	3.6	1	3.6	1	3.6
***Behaviour (BBI)***						
Normal	18	64.3	16	57.1	15	53.6
Non-normal	8	28.6	10	35.7	11	39.3
Not Available	2	7.1	2	7.1	2	7.1
***HADS DISTRESS***						
Mean	10.8		12.3		11.4	
Standard deviation	7.0		6.6		7.9	
Median	9.5		10.5		10.5	
Range	(0–27)		(3–26)		(0–30)	
% Above Cut-off	11	39%	12	43%	13	46%
***SeiQoL Index (SIS)***						
Mean	77.5		76.3		77.7	
Standard deviation	14.9		19.1		13.1	
Median	83.1		77.1		80.6	
IQR	(68.72–86.99)		(70.56–90.66)		(68.02–89.04)	
Range	(23.68–95.8)		(6.75–96.99)		(44.59–95.38)	

*MITOS* Milano-Torino Staging

*ECAS* Edinburgh Cognitive and Behavioural ALS Screen

*BBI* Beaumont Behavioural Inventory

*HADS* Hospital Anxiety and Depression Scale

*SeiQoL* Schedule for Individual Quality of Life

**Table 4:** Caregiver characteristics

	Caregivers		
	Interview 1	Interview 2	Interview 3
***Hours of care provided p/w***			
Mean	31.1	42.9	47.5
Standard deviation	34.2	43.6	44.8
Median	20	24.5	28
Range	(0–126)	(0–168)	(4–168)
***Caregiver Burden (ZBI)***			
Mean	22.3	25.6	27.1
Standard deviation	11.6	12.2	10.2
Median	20	25	27.5
Range	(7–52)	(5–57)	(6–49)
% Above Cut-off	(*n* = 8) 29%	(*n* = 14) 50%	(*n* = 16) 57%
***HADS DISTRESS***			
Mean	14.1	15.2	14.6
Standard deviation	5.9	7.6	7.1
Median	14	14	15.5
Range	(4–23)	(5–30)	(2–29)
% Above Cut-off	(*n* = 18) 64%	(*n* = 17) 61%	(*n* = 17) 61%
***SeiQoL Index (SIS)***			
Mean	75.3	72.8	71.6
Standard deviation	18.2	16.5	17.5
Median	80.64	72.69	77.94
IQR	(61.27–87.76)	(62.16–83.90)	(65.40–83.02)
Range	(31.12–97.7)	(31.24–98.05)	(29.07–96.24)

*ZBI* Zarit Burden Interview

*HADS* Hospital Anxiety and Depression Scale

*SeiQoL* Schedule for Individual Quality of Life

### Dyad characteristics

The dyads comprised 28 co-habiting spouse/partner couples. All patients had caregivers of the opposite sex i.e. 19 caregivers were female and 9 male (Tables 2, 3 and 4).

The majority of patients was male (68%), spinal onset (71%), with a mean age of 62 years at the first interview. There was a mean of 23.2 months (median 17.5) from diagnosis to the first interview. At baseline, MiToS functional staging criteria categorised 75% of patients at Stage 0 (no loss of independent function) as per standardised protocol [28] with 48% at stages 2 and 3 at the final interview. 32% of patients were assessed as cognitively impaired and 39% behaviourally impaired at the third interview.

The majority of caregivers was female (68%), mean age was 61 years at baseline, providing an average 31 h of care per week, rising to 47 at the 3rd interview. The mean burden score was 22.3 (sd 11.6) increasing to 27.1 (sd 10.2) at the third time point. A burden score (ZBI) of 24 or over is categorised as ‘high burden’ [35, 36], and 29, 50 and 57% of this caregiver cohort were in the ‘high burden’ category at the first, second and third interviews.

Repeated measures ANOVA showed the difference in burden scores was statistically significant over time (F (2, 54) = 3.770, *p* = .029), pairwise comparisons with Bonferroni correction revealed a significant difference in burden scores between baseline and the third interview (*p* = 0.030).

There was an overall negative trend in the association between caregiver quality of life and burden, i.e. higher levels of burden (ZBI) are associated with lower individual quality of life of caregivers. The negative association between caregiver quality of life (m = 71.61 sd = 17.49), and burden (m = 27.07, sd-10.05) was significant at the third interview (r = −.395 *p* = .037).

The mean level of psychological distress (HADS –T) was higher among caregivers than patients at each interview. The difference in mean levels of distress was not statistically significant over time for patients (F (2, 54) = 1.549 *p* = .222) or for caregivers (F (2, 54) = .457 *p* = .635).

Using the HADS –T cut-off score of 12 [32], patients mean HADS-T scores were marginally above the cut-off at the second interview, but psychological distress did not reach the cut off level at times 1 and 3; 39% of patients scored higher than the cut-off at the first interview, 43% at the second, and 46% at the third.

Mean levels of psychological distress for caregivers were above the cut-off point at all three interviews; and 64% of all caregivers were above the cut-off at the first interview, and 61% at the second and third.

### Individual quality of life

Twenty-eight caregiver-care recipient dyads completed the three interviews and SEIQoL-DW assessment on all occasions. The average time taken to complete an assessment was 16 min.

**Fig. 1:**
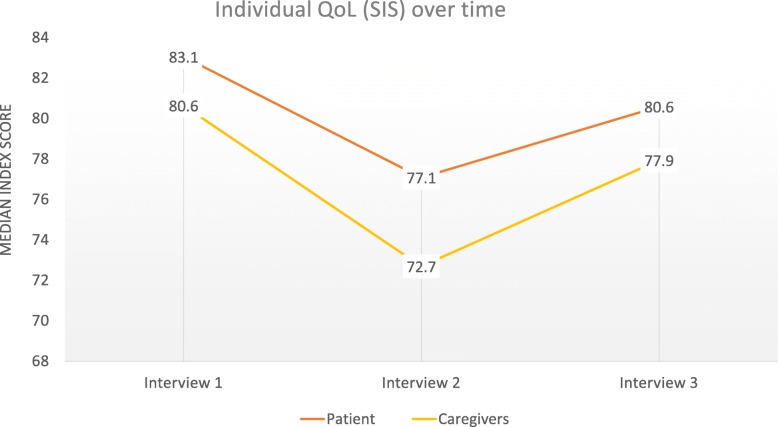
SEIQoL-DW Index Scores (SIS) – patient and caregiver

The median SEIQoL Index score (SIS) remained at a relatively high levels over the interview series (Fig. 1), and higher for patients than caregivers at each time point (Tables 3, 4 and Appendix B). IQoL was lowest at the second interview for both dyad partners. Results from a Friedman test showed that the differences in SIS over time were not statistically significant for patients χ^2^ (2) = .643, *p* = .725 or caregivers χ^2^ (2) = 3.071, *p* = .215.

There was an overall positive trend in the association between the IQol of patients and their caregivers i.e. higher SIS in patients is associated with higher SIS in caregivers. However, there was no statistically significant correlation at any of the interview timepoints 1, 2 or 3.

Considering the cognitive and behavioural status of patients (i.e. impairment/non-impairment) Mann Whitney U tests compared differences in median SIS over time.

For patients median SIS was not statistically different according to cognitive status: (U = 46, *P* = .321) time 1; (U = 62, *p* = .658) time 2; (U = 76; *P* = .797) time 3. Similarly differences in caregiver SIS depending on the cognitive status of the care recipient were not statistically significant (U = 42.5, *P* = .232) at time 1; (U = 63, *p* = .699) time 2; (U = 60; *P* = .280) time 3.

In relation to patients’ behavioural status, the difference in median SIS due to presence/ absence of impairment for either patients or caregivers did not reach statistical significance:. Patients: (U = 70, *P* = .912) at time 1; (U = 70, *p* = .598) time 2; (U = 70; *P* = .517) time 3; Caregiver SIS (U = 42.5, *P* = .101) at time 1; (U = 79, *p* = .856) time 2; (U = 81; *P* = .938) time 3.

### SeiQol index score (SIS) within dyads

**Fig. 2:**
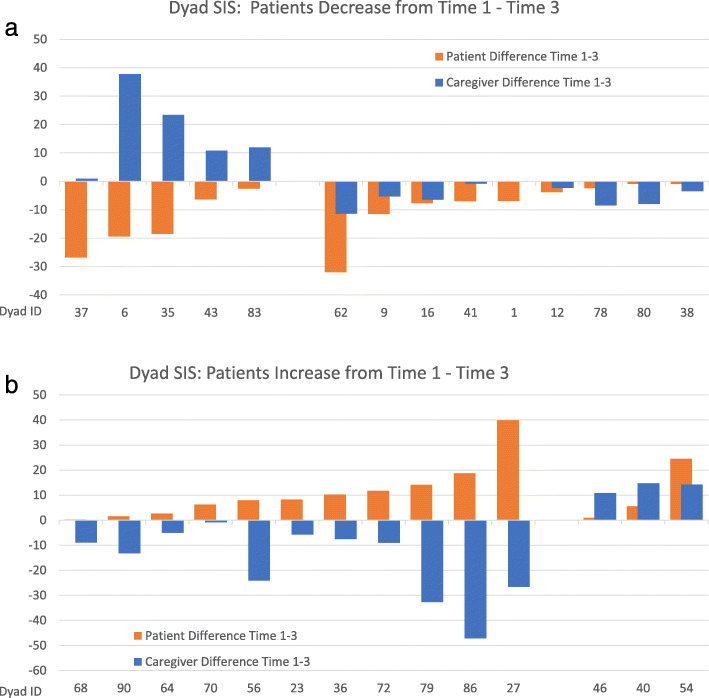
**a** illustrates that patient SIS decreased from the first to the third interview in half of the dyads in the study. In these 14 dyads, the SIS of caregivers also decreased in 9 but increased in 5 of them (e.g. Dyad IDs 37, 6, 35, 43, 83). **b** below shows the 14 dyads in which patient SIS increased from the first to the third interview. The caregiver quality of life increased in 3 but decreased for caregivers in 11 of the dyads in which patient SIS increased

The dynamic nature of individual quality of life (SIS) is illustrated in Fig. 2a and b showing the within-dyad variability of the changes in individual quality of life scores (median SIS) from the first to the third interview.

### Life areas contributing to individual quality of life

*As part of the SEIQoL assessment participants are asked:*

*“What are the five most important areas of your life at present – the things which make your life a relatively happy or sad one at the moment … the things that you feel determine the quality of your life?”*

Respondents named the areas of life that were important to them. Their responses were coded into 10 life domain categories (Table 5).

**Table 5:** Nominated Life Areas

Life area/Cue category	Sub categories
**1 Family**	Husband/wife, children, grandchildren, siblings, relationship, extended family
**2 Friends**	Friendship, supportive, networks
**3 Health/Wellbeing**	Own health (physical, mental), Other’s health (physical, mental)
**4 Finance**	Financial security, payment for treatment/carers/repairs, benefits
**5 Hobbies/Social activities**	Sports, travel, television, cinema, reading, art, cooking, arts and crafts
**6 Work**	Employment, tasks, colleagues, purpose
**7 Existential**	Hope, respect, freedom, independence, normality, time
**8 Faith/Religion**	Faith, God, spirituality, church
**9 Neighbours**	Local groups, community
**10 Other**	Pets/animals, nature, weather, home, environment, health services

The frequency with which these life domains were nominated are presented in Table 6.

**Table 6:** Frequency* of Nominated life areas

***Percent***^a^	Patient				Caregivers			
	Interview 1	Interview 2	Interview 3	Total	Interview 1	Interview 2	Interview 3	Total
Hobbies/Social	22.9	27.9	23.6	24.8	17.9	22.9	17.9	19.5
Family	22.1	29.3	30.0	27.1	33.6	30.0	33.6	32.4
Health	14.3	8.6	13.6	12.1	13.6	14.3	13.6	13.8
Friends	8.6	6.4	7.1	7.4	6.4	7.1	7.9	7.1
Finances	7.1	3.6	4.3	5.0	7.9	7.9	7.1	7.6
Other^b^	6.4	5.0	8.6	6.7	7.1	5.7	5.7	6.2
Existential	6.4	6.4	5.0	6.0	4.3	6.4	3.6	4.8
Work	6.4	5.7	2.1	4.8	6.4	5.0	6.4	6.0
Faith/Religion	3.6	5.0	2.1	3.6	2.1	0.0	2.9	1.7
Community	2.1	2.1	3.6	2.6	0.7	0.7	1.4	1.0

This table is ordered by the frequency of life areas nominated by patients at the first interview

^a^as % of all nominations

^b^e.g. pets, home, health services, weather, nature

Family and Hobbies/Social activities were nominated most often as contributors to quality of life. At the first interview family was mentioned more often by caregivers than patients. Hobbies and social activities were important contributors to patient quality of life over time and less so for their care partners. Health was nominated with similar frequency by both dyad elements although considerably less often than family and hobbies. In particular just 8% of all patients’ nominations were health-related at the second interview.

### Importance of nominated life areas relative to each other

*How important (are) the five areas of life you have nominated in relation to each other?*

Respondents indicated the importance of each of their 5 chosen life domains relative to each other (range 0–100). Table 8 details the mean relative importance of the life domains of patients and caregivers at each interview (Appendix C).

A Wilcoxon signed-rank test showed the relative importance of health for patients declined from the first to the third interview, and was statistically significant (*Z* = − 2.023, *p* = 0.043). This was the only significant result at (*p* < .05). Caution is advised regarding statistical significance due to small sample size.

**Fig. 3:**
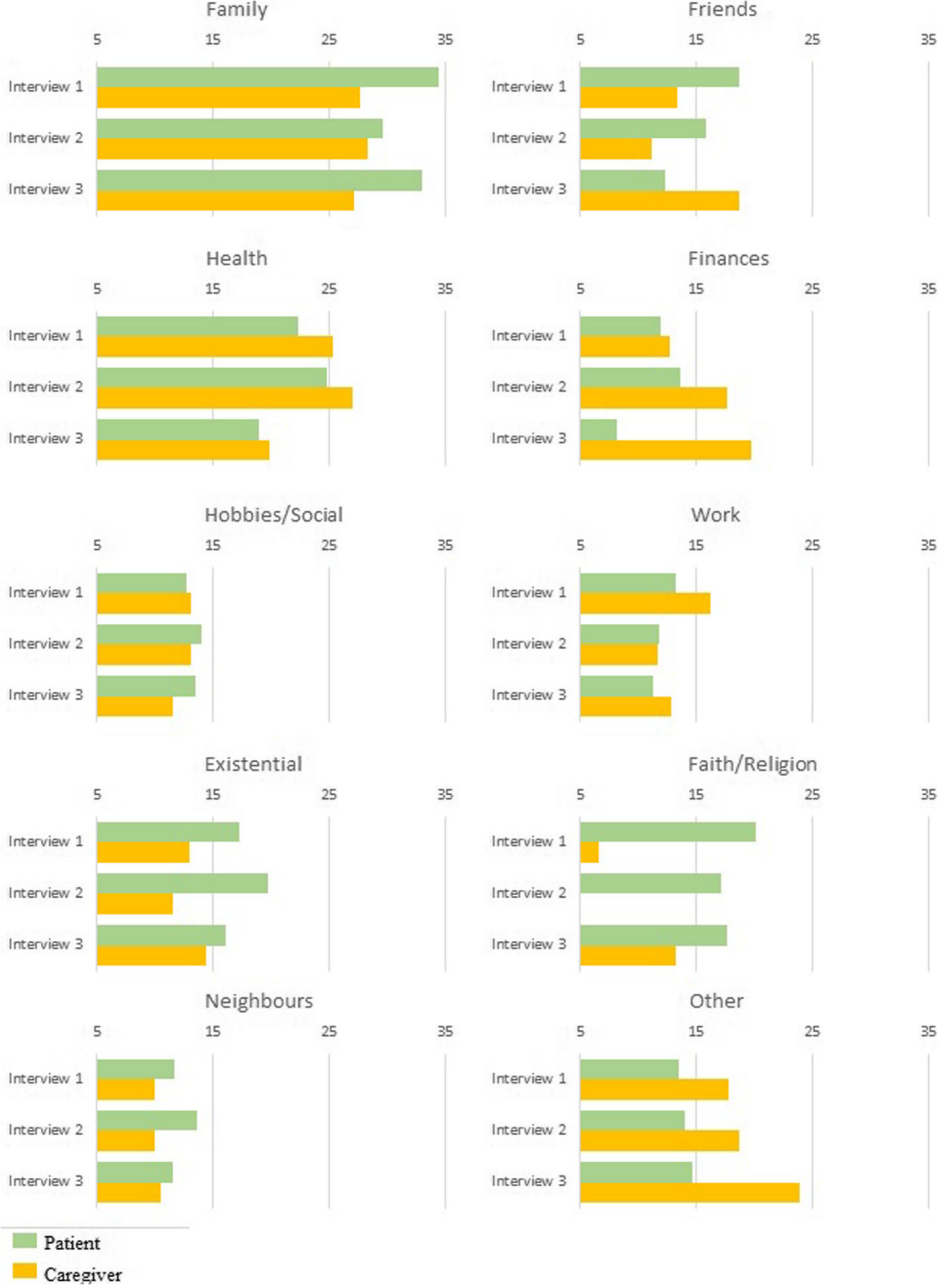
Mean relative importance over time

The nominated areas and assigned mean relative importance at each interview are illustrated in Fig. 3.

The *relative* importance of social activities for patients and caregivers was low at each interview. With time, the relative importance of health declined for patients; faith and existential factors became more important. Financial issues are assigned less importance by patients, and the converse for their caregivers over time. Factors such as animals, nature, health services and home environment categorised as ‘Other’ took on added importance for caregivers, especially at the third interview.

## Discussion

In the absence of curative treatments, quality of life is an important theoretical and clinical issue in ALS [41]. The experiences of caring for a person with ALS makes it an important issue for caregivers as well. Individual quality of life is meaningful in the real world context of an illness trajectory. SEIQoL-DW is suitable for use in a clinical setting when planning for patient care and its temporal application is valuable for understanding the complexity of individual QoL and its interaction with illness [11].

Quality of Life is both individual and relational in the illness scenario. People in relationships depend on one another, and illness makes interdependence explicit. Domains that would otherwise be private become open to relational scrutiny and negotiation [42]. Peoples’ evaluations of their QoL are made within the horizons of possibilities that they see for themselves. Perception of quality of life varies between individuals and is dynamic [43].

The choice of a QoL instrument is influenced by the concept of quality of life being used. SEIQoL-DW has been designed to assess individual quality of life and the domains that the participant perceives determine this. We suggest that a combination of standardised and individualised approaches is more likely to provide a broad and complementary approach to quality of life assessment. Person-perceived outcomes matter in clinical care and disease management. Self-identification of the factors which contribute to quality of life is one such outcome. The collection and analysis of both quantitative and qualitative data is ideal when addressing complex questions in health research and its translation to clinical practice.

This study explored individual quality of life of people with ALS and their partner caregivers over the course of 12–18 months. Patient IQoL (SIS) was higher than caregivers at each time point. The changes in SIS scores from the first to the third interview points to an independent dynamic within the dyads, as a significant number of caregivers had reduced quality of life over time in those dyads where patients’ quality of life increased. ALS/MND affects the functioning and well-being of both the patient and caregiver. The fluctuations of IQol within the couples over time, points to the need for further consideration of the interaction effect on individual functioning in a dyad context. The wellbeing of caregivers, and their ability to provide care is vital in supporting people with ALS to remain in their own homes.

Despite disease progression and with over one third of patients experiencing cognitive/behavioural impairment, it is noteworthy that individual quality of life scores (SIS) remained high for both patients and caregivers. Differences in SIS were not statistically significant according to the cognitive or behavioural status of the dyad, although average levels of psychological distress were higher for caregivers than care recipients. Caregiver burden also increased, with a significant difference between baseline and the third interview.

These findings support previous research.

In a systematic review De Wit et al. (2018) found that quality of life of caregivers worsens as the disease progresses and care demands increase [3].

Individual perception of quality of life for patients is preserved over the disease course despite physical decline [7, 9]. In a longitudinal study Gauthier et al. (2007) found stability in patient quality of life over time, which could reflect factors such as acceptance of and adjustment to the disease over time, denial or cognitive impairment [9].

SEIQoL-DW scores were lower for caregivers than for patients [44], which could be due to shifting patient expectations with disease progression.

Mean quality of life scores for patients increased and decreased for caregivers [9] the decrease was associated with caregivers’ impaired psychological health and physical symptoms.

The life domains nominated here were similar to previous work [45]. Neudert et al. (2004) found the QoL domains most often named in SEIQOL-DW were family, friends/social life, health, and profession.

If quality of life is conceptualised as being unique to individuals, it cannot be adequately assessed using standardised measures only. Individualised assessment facilitates the expression of important, non-predefined domains and why they are meaningful. Family, Hobbies/Social activities and Health were the most frequently nominated life areas contributing to quality of life for both patients and caregivers. Family included partners, immediate and extended family members, relationship and marriage. Social activities included walks, sport, art, music and cultural activities. Patients adjusted to functional limitations by focusing on what was still possible to do, rather what they are unable to: “*Choir is important to me now it’s an outlet. It’s replaced golf because I can’t play it, so that’s an area I do enjoy.”* The reconceptualization [16, 19] may partly explain the stability in IQoL over time. For caregivers, hobbies and social activities were ways to maintain their identity and get respite from care duties. Health was nominated with similar frequency by both patients and caregivers, describing their own health and the health of others. Caregivers described their need to be healthy and able to provide care to the patient and other family members: “*you have to look after yourself. I won’t be any use to xx if I wasn’t healthy. I’d like to have time to give to other family members in the future”.*

When asked to rate their nominated life areas relative to each other, the importance of family was consistent over time. Friendship and non-family social engagements became more important for caregivers: “*close friends you can have a chat with. You’re comfortable with them. They’re outside the family”.* Similarly, among caregivers the significance of financial issues assumed increasing importance. Reduced or absent incomes and the material costs associated with caregiving become apparent. The Health domain was assigned lesser importance among both dyad elements from baseline to the third interview. Life domains are re-evaluated and reconfigured as patients and their care partners adapt to changing functional, material and emotional contexts.

The findings from this study have implications for clinical management and health care practice. IQoL can be monitored across the disease trajectory, and allows a closer examination of what the individual self-identifies as important to him/her. It is important to understand the determinants of quality of life the composition of which is self-defined. People with compromised cognitive and behavioural status and their informal caregivers maintained a good quality of life. Palliative and supportive care services should aim to support the domains identified to help maintain life quality as long as is practicable. Routine clinical evaluation of IQoL could facilitate communication between patient, caregiver and health care professionals, to guide care planning informed by the illness experiences. Multidisciplinary clinics should have staff available to address psychosocial aspects of patient and caregiver well-being as part of the routine clinical encounters.

### Strengths and limitations

This study is a unique integrated exploration of individual quality of life, for patient and caregiver dyads in neurodegeneration over time. The findings are applicable to attenders at a multidisciplinary clinic. Supports provided in a multidisciplinary clinic may influence expectations and quality of life.

A strength of this study is its mixed methods approach, and integrated analyses. Both qualitative and quantitative data were collected through a single assessment instrument. The data were analysed quantitatively, with quantification of the descriptive content and contextualised qualitatively. The use of individual measures and standardized questionnaires captures complementary facets considered important in life [8]. However, further research is needed to better understand how to maintain good QoL despite changing circumstances due to the characteristics of the disease, and to explore within-dyad interaction.

Deteriorating illness, research fatigue, and non-participation of either dyadic partner resulted in reducing numbers completing the IQoL assessment over the course of the interview series. Participant attrition across the interviews could have introduced bias and it may be the disease is less burdensome in this cohort. Larger sample size would allow for assessment of statistically significant differences.

Although it was not the case in this study, the completion of SEIQoL-DW assessment tool may be difficult for some people with severe impairment to complete. The SEIQoL-DW is of value in identifying factors which contribute to the well-being of an individual with ALS, rather than used to measure of the QoL of groups [33].

This was a spousal/partner dyadic cohort, and findings may differ by relationship type between care recipient and caregiver. The influence of cognitive and behavioural impairment for patients and caregivers did not impact of self-reported quality of life. This is at variance with the observation that caregiver burden is impacted by severe behavioural change among patients. This dichotomy should be investigated in larger samples.

## Conclusion

Quality of life is relatively stable despite disease progression for this dyadic cohort. The domains nominated as contributing to quality of life are consistent over time, with both divergence and similarities among patients and caregivers. The importance of domains relative to each other varies by dyad partner and with time.

Individual quality of life as a mixed method outcome measure provides unique information for a variety of interventions tailored to address patients’ and caregivers’ concerns. The assessment of individual Qol is an approach that moves away from generic or disease-specific measures of health status, placing the emphasis instead on the unique situation and perspective of each person.

Individual quality of life should be assessed in conjunction with other disease-specific or health related quality of life measures. Single global quality of life scores are useful for comparative purposes however they may mask the different factors which contribute to quality of life at the individual level.

This study has illustrated the importance of family, the continuing importance of social activities for patients and the increasing importance of friends and financial issues for their partner caregivers. A disease course has an objective dimension however the experience of illness is subjective and dynamic. We propose that IQoL, and the self-defined contributors to it should be assessed and monitored as part of routine clinical care. This insight into patient and caregiver wellbeing should be elicited early in the disease trajectory and monitored over time by health care professionals, facilitating person-centred care. IQoL assessments should form an important base for health care providers when planning for clinical management and care. The importance of support for patients and caregivers provided on an individual basis, and in dyads, across the disease course should be recognised.

## Figures and Tables

**Fig. 4:**
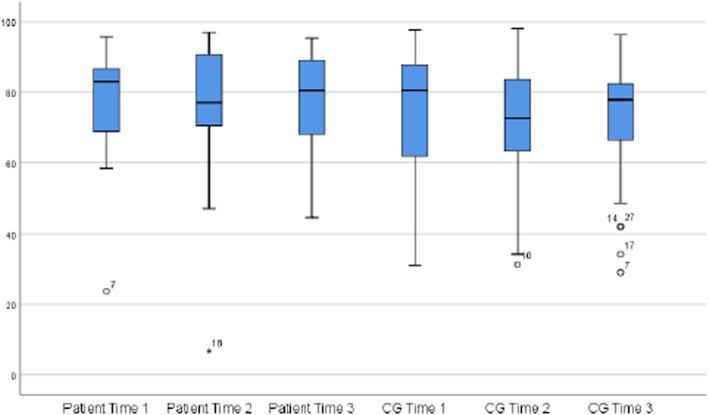
SIS scores for patients and their caregivers. The global SIS scores for patients and their caregivers across the 3 interviews are shown in the boxplots below. There are some outliers, however we do not see these as ‘bad’ data points, but rather represent the individual’s perception of his/her quality of life, and as such are included in the analysis

## Data Availability

The material generated and analysed during the current study cannot be made publicly available for reasons of privacy and confidentiality (Beaumont Hospital Medical Research Ethics Committee). However, access to de-identified datasets can be made available on request to Mr. Mark Heverin mark.heverin@tcd.ie

## Appendix

### Appendix A

**Table 7:** Administration of SEIQoL-DW

***Applying the SEIQoL-DW (Hickey*** **et al** ***1996)*** [18]	
The SEIQoL-DW is administered in a standardised semi-structured interview in three steps.	
(1) **Cue elicitation***-“What are the five most important aspects of your life at the moment?”* The individual is asked to name the five areas of life (cues) which are most important to the overall quality of his or her life.	
(2) **Determining current status on each cue***-“How would you rate yourself on each of these areas at the moment, on a scale from the worst possible to the best possible?”* The respondent rates current status against a vertical visual analogue scale labelled at the upper extremity by “as good as could possibly be” and at the lower extremity by “as bad as could possibly be.” These ratings are recorded in the form of a bar chart. The possible score range for each cue level is 0 to 100.	
(3) **Quantification of relative weighting of each cue**-“*How do the five areas compare in importance to each other?”* This final step involves quantifying the relative contribution of each elicited cue to the judgment of overall quality of life using the direct weighting instrument described above. The total value of all five weights sums to 100.	
The SEIQoL-DW allows measurement of quality of life to be completely individualised.	
To present information as grouped data, for making group comparisons, it is possible to derive a single index from the data-the global quality of life score. This is calculated by multiplying the individual’s current self-rating on each cue by the corresponding cue weight and summing the products across the five cues. This global quality of life score can range from 0 to 100; it is a continuous measure which can be subjected to parametric statistical analyses	

### Appendix C

**Table 8:** Table below shows the mean relative importance of the life domains of patients and caregivers

Life area/Cue category	Patient			Caregivers		
	Interview 1	Interview 2	Interview 3	Interview 1	Interview 2	Interview 3
1. **Family**	34.4	29.6	33.0	27.6	28.3	27.2
2. **Friends**	18.7	15.9	12.3	13.3	11.2	18.7
3. **Health/Wellbeing**	22.4	24.8	19.0	25.4	27.0	19.8
4. **Finance**	12.0	13.6	8.2	12.7	17.6	19.7
5. **Hobbies/Social activities**	12.8	14.0	13.5	13.1	13.1	11.5
6. **Work**	13.2	11.9	11.3	16.2	11.7	12.9
7. **Existential**	17.3	19.8	16.1	13.0	11.6	14.4
8. **Faith/Religion**	20.2	17.1	17.7	6.7	0.0	13.3
9. **Neighbours**	11.7	13.7	11.6	10.0	10.0	10.5
10. **Other**	13.6	14.0	14.8	17.8	18.8	23.9

## Abbreviations

ALS: Amyotrophic Lateral Sclerosis
BBI: Beaumont Behavioural Inventory
ECAS: Edinburgh Cognitive and Behavioural ALS Screen
HADS: Hospital Anxiety and Depression Scale
IQoL: Individual Quality of Life
MiToS: Milano-Torino Staging
MND: Motor neurone disease
QoL: Quality of Life
SEIQoL-DW: Schedule for the Evaluation of Individual Quality of Life
SIS: SEIQoL Index QoL Score
ZBI: Zarit Burden Interview
